# Remediation of suspended solids and turbidity by improved settling tank design in a small‐scale, free‐standing toilet system using recycled blackwater

**DOI:** 10.1111/wej.12369

**Published:** 2018-09-17

**Authors:** Brian T. Hawkins, Katelyn L. Sellgren, Enzo Cellini, Ethan J. D. Klem, Tess Rogers, Brendon J. Lynch, Jeffrey R. Piascik, Brian R. Stoner

**Affiliations:** ^1^ RTI International Research Triangle Park NC USA; ^2^ Department of Electrical and Computer Engineering Duke University Durham NC USA; ^3^ Center for WaSH‐AID Duke University Durham NC USA; ^4^ Micross Components Research Triangle Park NC USA; ^5^ Triangle Environmental Health Initiative Durham NC USA; ^6^ Biomass Controls Durham NC USA

**Keywords:** decentralized waste water treatment, electrochemistry, sustainability, water reuse

## Abstract

Our research is focused on the development of decentralized waste water treatment technologies enabling onsite water reuse. Accumulation of solids with recycling of treated blackwater increases the energy required for disinfection with an electrochemical process. We hypothesized that improving the preprocess settling of blackwater by increasing the tortuosity of the liquid flow path would reduce this energy demand by reducing particle‐associated chemical oxygen demand (COD). This approach successfully reduced the total suspended solids and turbidity in the process liquid accumulated per user‐day equivalent. A modest reduction in the apparent steady‐state accumulation of COD was also observed, likely because of the retention of COD associated with larger particles in the settling tanks. Interestingly, these improvements did not improve the energy efficiency of the electrochemical disinfection process, as predicted. These observations suggest that improving the energy efficiency of electrochemical disinfection will require remediation of dissolved COD.

## 1 Introduction

Proper sanitation is indispensable for human health. The lack of access to improved sanitation facilities amongst approximately 1/3 of the global population (WHO/UNICEF, 2014) constitutes a tremendous impediment to reducing global poverty. However, improving sanitation in low‐resource settings is not simply a matter of deploying existing technology, but rather requires designs that take the limitations of material and infrastructure into account (Niemeier et al., 2014). Supplying clean water requires both a consistent source of fresh water and the ability to effectively and sustainably treat waste water, which in turn requires considerable investments in energy and water infrastructures, both of which present challenges to deployment in lower income countries.

We are developing a free‐standing toilet system that is designed to separate liquid and solid waste, drying the solids for use as a combustible fuel to drive thermoelectric devices and disinfecting the liquid with an electrochemical process enabling safe onsite reuse (Stokes et al., 2014; Rogers et al., 2016; Sellgren et al., 2017). The ultimate goal is an integrated system that requires no addition of external water or power; however, the energy required to achieve effective and reliable disinfection of blackwater using the electrochemical process currently exceeds what can be generated onsite. Furthermore, repeated recycling of treated blackwater leads to accumulation of dissolved and suspended solids in the process liquid that is associated with an increase in the amount of energy required to achieve full disinfection (Hawkins et al., 2017).

Oxidant contact time (and thus energy) required for electrochemically produced oxidant inactivation of *Escherichia coli* in food processing waste waters increases with increased chemical oxygen demand (COD) of the process liquid (Lopez‐Galvez et al., 2012). As much as 60% of COD in concentrated blackwater is associated with particles large enough to potentially be removed by settling and/or practical filtration (Hocaoglu and Orhon, 2013). Therefore, we hypothesized that by improving the preprocess settling of blackwater in our system, we could both improve the appearance of the process liquid and potentially reduce the energy required for electrochemical disinfection by reducing particle‐associated COD.

**Figure 1:**
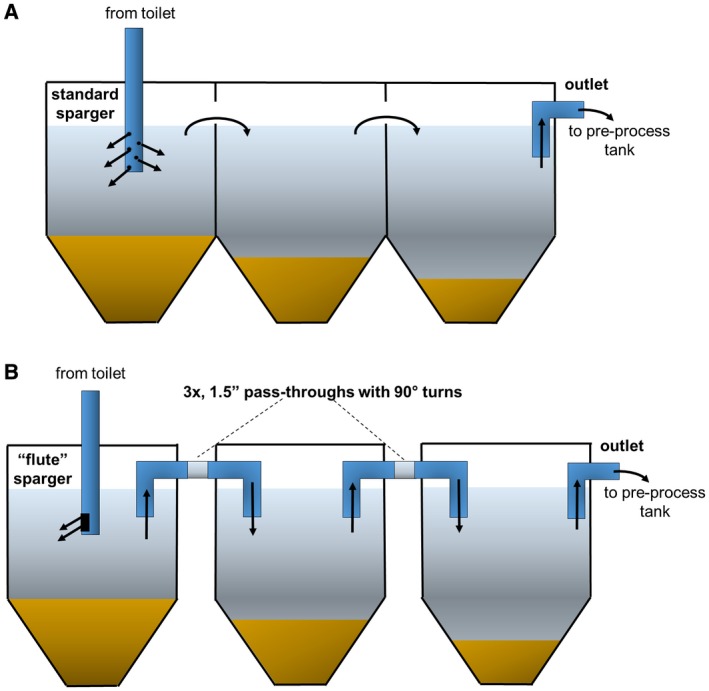
Settling tank designs. (A) The ‘simple’ tank design used in the original liquid disinfection system prototype. The system consists of three, 12‐L polyethylene containers with 54° sloped bottoms, connected in series by plastic thermal welding via ~1″ × 4″ openings cut near the tops of the tanks, yielding effective volumes of ~10 L each. The sparger is perforated to allow diffuse liquid flow in all lateral directions. The final tank is connected via a gravity feed to a polyethylene preprocess holding tank. (B) The ‘enhanced’ tank design uses the same polyethylene tanks, but replaces the direct connections by thermal welding with 3, 1.5″ PVC pass through pipes between each tank with two 90° turns. The perforated sparger is replaced with a flute‐style sparger with a single, 3 × 4‐cm rectangular cutout directed away from the outlet and the final tank is connected via a gravity feed to a polyethylene preprocess holding tank. [Colour figure can be viewed at wileyonlinelibrary.com]

Our system is designed to handle up to 10 users per day in high population density settings, which limits the physical footprint of the overall system. As such, large settling tanks enabling very long retention times are not practical. Our previous prototype system made use of a compact, three‐tank design with a total effective volume of 30 L to settle particulates out of the process liquid prior to electrochemical treatment (Fig. 1A). In the studies described here, we sought to improve the settling and retention of particles in our preprocess settling tanks by increasing the tortuosity of the liquid flow path and minimizing the disturbance of previously settled solids (Fig. 1B), whilst maintaining the small footprint and short retention time necessitated by spatial constraints of our system. We compared the resulting process liquid characteristics between the different settling tank designs, and the impact on disinfection energy demand of the electrochemical treatment process.

## 2 Experimental methods

Our prototype liquid disinfection system and the sample collection and laboratory procedures used in this study have been described in detail previously (Hawkins et al., 2017; Sellgren et al., 2017). Collection procedures for human urine and feces from healthy volunteers were approved by RTI’s institutional review board. Samples were flushed into the prototype toilet (initially charged with tap water) at rates intended to approximate per person urine and faecal production rates of 1.5 L and 130 g per day, respectively (Rose et al., 2015). Total urine volumes ($\sum^{}v_{\text{urine}\;}$) and faecal masses ($\sum^{}m_{\text{feces}\;}$) flushed into the system were continuously logged and used to calculate user‐day equivalents (UDE) by the following equation:

(1) $$\text{UDE}=\frac{\frac{\sum^{}v_{\text{urine}\;}}{1.5\;\text{L}}\;+\;\frac{\sum^{}m_{\text{feces}\;}}{130\;\text{g}}}{2}$$

UDE were used throughout the study to index all data collected to the estimated ‘usage’ of the system from startup. Processed (disinfected) liquid waste was recycled through the system as flush liquid for subsequent flush cycles, with excess processed liquid (equivalent in volume to urine flushed into the system) discharged.

Liquid waste was treated in 30‐L batches with a two‐stage electrochemical process (60–120 min at 24 Vdc followed by 90–180 min at 32 Vdc) using a commercially available electrochemical cell (Hayward Salt&Swim 3C). The electrochemical cell consisted of 13 dual‐sided mixed‐metal oxide (MMO) electrodes, 64 cm^2^ in area each, separated by a 3‐mm gaps. Current through the electrochemical cell was monitored with a Mastech MS2138R AC/DC clamp meter, and electrochemical energy per volume of process liquid used at time *n* (*E_n_*) was estimated by:

(2) $$E_{n}=\;\;\frac{\;V\int_{0}^{n}I\left(t\right)dt}{v}$$

where *V* is the voltage, *I* is the current, *ν* is the volume being treated. The integral of current with time was estimated by the trapezoid method.

Conductivity was measured using a Myron L 6PFCE Ultrameter II (Myron L Company, Carlsbad CA). COD was measured using the Reactor Digestion Method (HACH method 8000) using a HACH DRB200 reactor and a HACH DR 900 colorimeter (HACH, Loveland, CO). Samples were run according the manufacturer’s instructions; briefly, 2 mL of sample were added to a digestion vial, mixed well and reacted at 165°C for 2 h. After cooling to room temperature absorbance was measured using program 435 COD HR. Samples with readings out of range were diluted using DI H_2_O. Blanks consisted of 2 mL of DI H_2_O. Turbidity was measured with a HACH 2100Q IS following the manufacturer’s instructions. Total suspended solids (TSS) were determined using Standard Method 2540D. This was accomplished by weighing liquid samples (~10 mL) in tared tubes, washing the samples through tared 0.7‐μm filter paper (Fisher) and weighing the filter papers after drying at 103–105°C. Microbes were enumerated with a most probable number (MPN) method as previously described (Sellgren et al., 2017). Disinfection energy thresholds for MPN = 5/mL for each disinfection run were interpolated from the plots of log (MPN) versus *E_n_* as previously described (Hawkins et al., 2017). Statistical calculations were performed with GraphPad Prism v7.01.

## 3 Results and discussion

**Figure 2:**
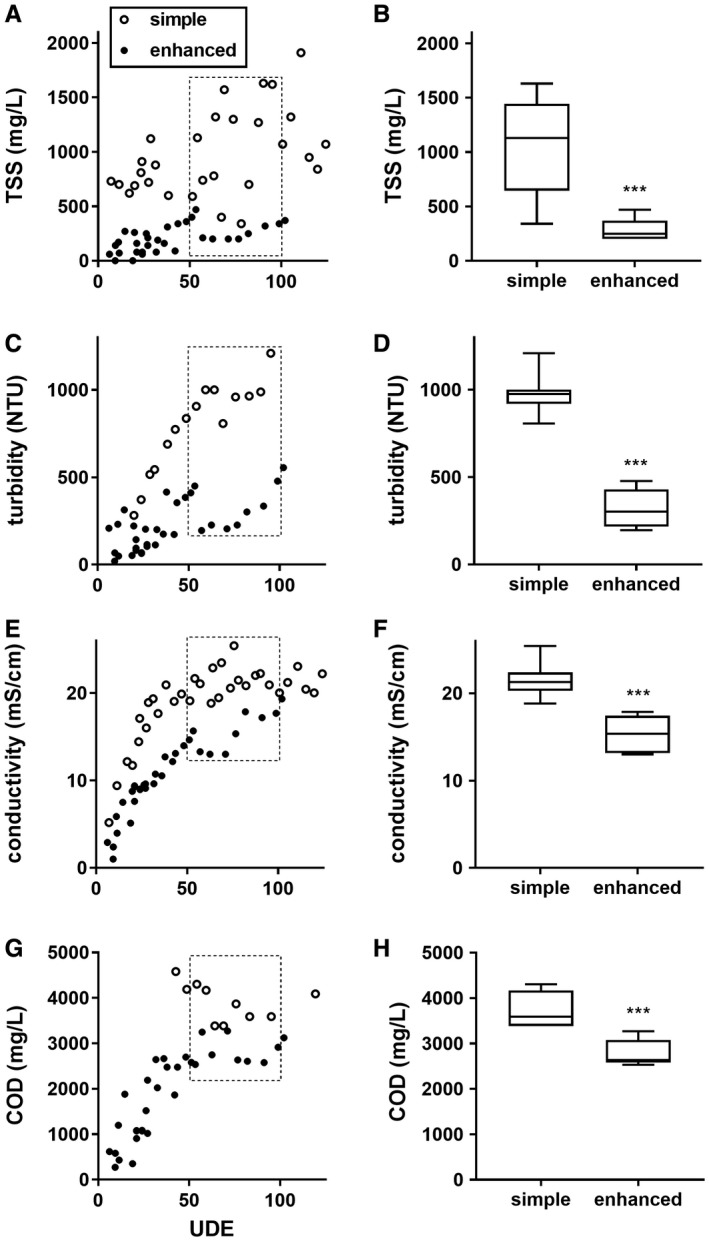
Effects of enhanced settling tank design on process liquid characteristics. All data were taken from samples drawn from the process tank prior to electrochemical treatment. (A, C, E and G) Scatter plots of parameters indicated versus user‐day equivalents (UDE) as determined by Equation (1) . (B, D, F and H) Box and whisker plots of the data points in the corresponding scatter plots collected between 50 and 100 UDE (indicated by the dotted line box in the corresponding scatter plots). Line indicates the median, box indicates the 25th and 75th percentiles, error bars indicate the minimum and maximum. ***P < 0.001, determined by unpaired two‐tailed *t*‐test.

Flush cycles were performed for each set of tests over several weeks as previously described (Hawkins et al., 2017). Because the composition and amounts of faecal and urine inputs to the system were variable, UDE were calculated to compare data collected in systems at different times. On a UDE basis, process liquid from the enhanced settling system had consistently lower TSS and turbidity throughout the period of testing (up to 120 UDE) (Fig. 2A and C). The conductivity of the process liquid approached the same ‘steady state’ range (18–23 mS/cm) in both systems, but took nearly twice as many UDE to reach steady state with the enhanced settling system (Fig. 2E). COD testing was not yet implemented in the earliest tests (<50 UDE) of the simple settling system; however, comparison of data from the same UDE range in both systems (50–100 UDE) indicated that there was a modest improvement (26% reduction) in accumulation of COD with the enhanced settling system (Fig. 2G and H). Similarly, comparison of all parameters in the 50–100 UDE range indicated consistent and statistically significant improvements in process liquid quality with the enhanced settling system, with the largest changes seen in TSS and turbidity (Fig. 2B and D), indicating that the enhanced settling system improved retention of particulates without additional filtration.

Removal of TSS in waste water treatment depends on a number of factors, including the speed at which water moves through the settling tanks, the hydraulic retention time (HRT) and the geometry of the system (Patziger and Kiss, 2016). In our settling systems, the HRT is highly variable because the inlet comes directly from a toilet, Flushes under lab conditions were typically performed in batches (approximately 1 flush every 5 min), leading to HRT as low as ~1.25 h. Thus, our system relies heavily on its geometry to slow the overflow rate and minimize the kinetic energy that previously settled solids experience.

The physical configuration of our system precluded sampling of influent in these studies, which makes a direct comparison of our system to other systems in terms of overall TSS removal difficult. However, we know that our influent consisted entirely of urine, flush liquid (recycled blackwater) and the faecal solids that passed through the solid‐liquid separator. The rates at which we flush urine and feces into the system result in a net input to the system of ~22 g feces per L of total liquid (Hawkins et al., 2017), and our solid‐liquid separator typically retains 80–90% of the faecal mass flushed and diverts it to a solid treatment system. Thus, we estimate that between 2200 and 4400 mg/L of faecal solids enters the liquid treatment system, and that the upper bound on potential suspended solids in our influent likely falls within this range. We also know from measurements in the original settling tank design that at steady state the TSS of our settling tank effluent was frequently >1000 mg/L (Fig. 2A and B), meaning that the influent TSS was at least 1000 mg/L and likely higher. Therefore, even with a conservative estimate of 1500 mg/L as a typical influent TSS, the enhanced settling system removed 86 ± 8% of influent TSS. This compares favourably with a number of systems, including a pilot‐scale anaerobic baffled reactor recently reported by Hahn and Figueroa (2015) which reduced TSS by 83 ± 10% with a much larger total capacity (1000 L).

It is possible this estimate is too optimistic; for example, improved retention of TSS in the enhanced settling system also means that the TSS in the recycled flush liquid (which makes up 75% of the influent by volume) was lower than it was with the original design. However, even if we revise our estimated influent TSS down to 500 mg/L we still saw 58 ± 23% TSS removal in the enhanced settling system, which is comparable to the reported performance of modern primary clarifiers in waste water treatment plants (Patziger and Kiss, 2015).

In a separate series of filtration experiments, we found that ~50% of COD in blackwater from the preprocess tank was removed by a 0.7‐μm filter (the same filter size we use to determine TSS), suggesting that this fraction of COD is associated with suspended solids, in good agreement with previous reports (Levine et al., 1985; Hocaoglu and Orhon, 2013). We observed a 72% decrease in process liquid TSS with the enhanced settling system compared to the simple system (Fig. 2B). Thus, the 26% reduction in COD we observed with the enhanced settling system (Fig. 2H) is only slightly less than the ~36% reduction we would predict based on the particle size distribution of COD in blackwater from our system.

**Figure 3:**
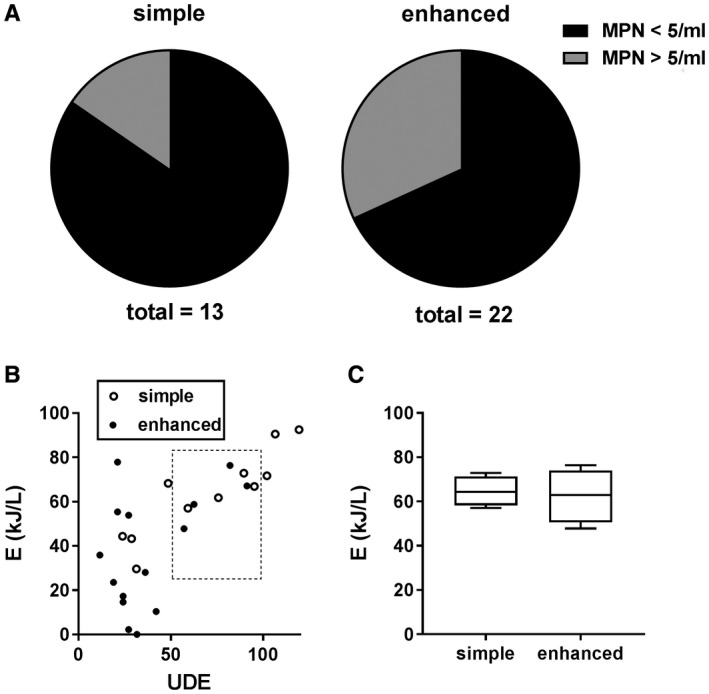
Disinfection efficacy and energy efficiency. (A) Pie charts comparing the proportion of trials in which the disinfection threshold [most probable number (MPN) < 5/mL] was reached in each system. (B) Scatter plot of energy required to achieve the disinfection threshold in all successful trials in both simple and enhanced systems versus user‐day equivalents (UDE). (C) Box and whisker plot of disinfection energies for all successful trials conducted between 50 and 100 UDE (indicated by the dotted line box in panel B). Line indicates the median, box indicates the 25th and 75th percentiles, error bars indicate the minimum and maximum.

Because the system is intended to treat waste water for reuse as flush and/or hand wash water, a stringent disinfection threshold is required to ensure user safety. As previously described (Hawkins et al., 2017), the process liquid typically had preprocess microbe counts (MPN) of ~10^7^/mL. In all trials, MPN were reduced to below 10^2^/mL, though disinfection to below 5/mL is the threshold we have set for ‘complete’ disinfection, in line with discharge limits in India (Ministry of Environment and Forests, India, [Link]) where field tests are underway. This threshold was met in 11 of 13 trials in the simple settling system and in 14 of 22 trials in the enhanced settling system (Fig. 3A). Comparison of the disinfection data between systems with the Fisher’s exact test indicated that the difference in disinfection efficacy (defined as the proportion of trials meeting the 5/mL threshold) between systems was not significant (*P* = 0.4311). Energy required to meet the 5/mL threshold increased with increasing UDE in both systems (Fig. 3B), and comparison of disinfection energies from successful trials in the 50–100 UDE range showed no difference in the energy requirement for full disinfection between systems (Fig. 3C). Thus, the improvements in process liquid characteristics realized by the enhanced settling system were not associated with improved efficacy or efficiency of the electrochemical disinfection process.

Taken together, these data indicate that whilst up to 50% of COD in blackwater is associated with larger particulates that can be partially remediated by improved preprocess settling, the COD associated with smaller particles and soluble species likely represent the biggest energy drag on the electrochemical disinfection process. It is possible that particle‐associated COD is partially shielded from oxidants in the bulk solution, making it resistant to removal by oxidation but also making it relatively unavailable to compete with microbes for oxidants; thus, its removal has minimal impact on the energy efficiency of electrochemical disinfection. High levels of soluble COD in the bulk solution, on the other hand, present many more opportunities for oxidants to be chemically neutralized rather than kill microbes, particularly when microbe counts are very low (<10^2^/mL), leading to the ‘long tail’ in the disinfection curve observed (Hawkins et al., 2017). Thus, substantial remediation of soluble COD in the process liquid is likely required to increase the energy efficiency of the electrochemical process for complete disinfection of blackwater.

## 4 Conclusions

1. Increasing the tortuosity of the liquid flow path through the settling system improves retention of particulates, as demonstrated by substantial reductions in TSS and turbidity in the process liquid.
2. These observations, together with the determination that ~50% of COD in blackwater from this system is found in soluble species and in particles too small to be readily settled, suggest that focusing on remediation of soluble components of COD is necessary to achieve a significant decrease in electrochemical disinfection energy demand.
